# The aquaporin 5 -1364A/C promoter polymorphism impacts on resolution of acute kidney injury in pneumonia evoked ARDS

**DOI:** 10.1371/journal.pone.0208582

**Published:** 2018-12-05

**Authors:** Tim Rahmel, Hartmuth Nowak, Katharina Rump, Winfried Siffert, Jürgen Peters, Michael Adamzik

**Affiliations:** 1 Klinik für Anästhesiologie, Intensivmedizin und Schmerztherapie, Universitätsklinikum Knappschaftskrankenhaus Bochum,Bochum, Germany; 2 Institut für Pharmakogenetik, Universität Duisburg-Essen & Universitätsklinikum Essen, Essen, Germany; 3 linik für Anästhesiologie und Intensivmedizin, Universität Duisburg-Essen & Universitätsklinikum Essen, Essen, Germany; University of Sao Paulo Medical School, BRAZIL

## Abstract

**Background:**

Aquaporin 5 (*AQP5)* expression impacts on cellular water transport, renal function but also on key mechanisms of inflammation and immune cell migration that prevail in sepsis and ARDS. Thus, the functionally relevant *AQP5* -1364A/C promoter single nucleotide polymorphism could impact on the development and resolution of acute kidney injury (AKI). Accordingly, we tested the hypothesis that the *AQP5* promoter -1364A/C polymorphism is associated with AKI in patients suffering from pneumonia evoked ARDS.

**Methods:**

This prospective study included 136 adult patients of Caucasian ethnicity with bacterially evoked pneumonia resulting in ARDS. Blood sampling was performed within 24 hours of ICU admission and patients were genotyped for the *AQP5* promoter -1364A/C single nucleotide polymorphism. The development of an AKI and the cumulative net fluid balance was described over a 30-day observation period and compared between the AA and AC/CC genotypes, and between survivors and non-survivors.

**Results:**

Incidence of an AKI upon admission did not differ in AA (58%) and AC/CC genotype carriers (60%; p = 0.791). However, on day 30, homozygous AA genotypes (57%) showed an increased prevalence of AKI compared to AC/CC genotypes (24%; p = 0.001). Furthermore, the AA genotype proved to be a strong, independent risk factor for predicting AKI persistence (odds-ratio: 3.35; 95%-CI: 1.2–9.0; p = 0.017). While a negative cumulative fluid balance was associated with increased survival (p = 0.001) the *AQP5* promoter polymorphism had no impact on net fluid balance (p = 0.96).

**Conclusions:**

In pneumonia evoked ARDS, the AA genotype of the *AQP5* promoter polymorphism is associated with a decreased recovery rate from AKI and this is independent of fluid balance. Consequently, the role of *AQP5* in influencing AKI likely rests in factors other than fluid balance.

## Introduction

Following advances in acute respiratory distress syndrome (ARDS) treatment including maintenance of gas exchange by extracorporeal membrane oxygenation (ECMO), many patients do not die from hypoxemia but from related organ failure [1, 2]. In ARDS, acute kidney injury (AKI) occurs frequently [3, 4], and despite renal replacement therapies, the morbidity and mortality associated with AKI remains unacceptably high [4, 5]. In fact, the duration and severity of an AKI is recognized as important risk factors for adverse outcomes [6]. Therefore, patients are stratified in distinct phenotypes of an AKI associated with recovery or with poor prognosis [6, 7].

Some of the variability regarding the recovery from an AKI may be influenced by genetic variations. A promising candidate gene for investigation of AKI in the context of ARDS is the water channel aquaporin 5 (*AQP5*). *AQP5* is associated not only with transcellular and renal fluid transport [8, 9], cell proliferation [10], but also with key mechanisms of inflammation, including immune cell migration [11, 12]. In humans, an altered *AQP5* expression is linked with a common single nucleotide polymorphism (SNP; -1364A/C; rs3759129) in the *AQP5* gene promoter [13]. Substitution of cytosine for adenosine at position -1364 is associated with decreased *AQP5* expression [13] and had significant impact on survival in patients suffering from sepsis [14]. Thus, an altered *AQP5* expression due to the *AQP5* -1364A/C promoter SNP could impact on the development, duration and recovery of an AKI potentially explaining the higher mortality of AA genotypes associated with an increased *AQP5* expression [14].

Accordingly, we tested the hypothesis that the *AQP5* -1364A/C promoter polymorphism is associated with the duration and recovery of an acute kidney injury in patients with ARDS.

## Material and methods

This study was reviewed and approved by the Ethics Committee of the Medical Faculty of the University of Duisburg-Essen (protocol no. 01-97-1697) and written informed consent was obtained from patients or their guardians. Patients admitted to the intensive care unit (ICU) of University of Duisburg-Essen Medical School were eligible for enrollment if ARDS was evoked by a bacterial pneumonia, their condition fulfilled the criteria of the Berlin definition [15, 16], had received ventilation for less than 7 days and had no previous history of ARDS. Exclusion criteria were an age less than 18 years, mechanical ventilation for 7 days or longer, pregnancy, long-term chronic respiratory insufficiency treated with oxygen therapy or non-invasive ventilation, severe chronic kidney disease (KDIGO category ≥ G4) and a decision to withhold or withdraw life sustaining therapies on the day of study inclusion.

One hundred thirty-six patients of Caucasian ethnicity with ARDS (79 males (58%), 57 females (42%), mean age: 43.7 years SD ± 15.1) were prospectively included. Briefly, ARDS was evoked in 110 cases (81%) by bacterial pneumonia and in 26 cases (19%) by a primary extrapulmonary sepsis with a secondary bacterial pneumonia leading to ARDS. Bacterial infection was proven by positive pathogen detection from lung and/or blood cultures.

Clinical and demographic data upon study entry, including pre-existing morbidities, Sepsis-related Organ Failure Assessment Score, net fluid balance per day, necessity for continuous hemofiltration/-dialysis, PaO2/FiO2 ratio (Horowitz index), establishment of extracorporeal membrane oxygenation therapy, pulmonary function variables (mean airway pressure, positive end-expiratory pressure, compliance, pulmonary arterial pressure, pulmonary vascular resistance), medications and dosages of vasoactive drugs and blood chemistry values were recorded. All patients were categorized daily according to the degree of acute kidney injury as described in the recent KDIGO international guideline [17] on the basis of serum creatinine concentration and urine output. The worse of both values was applied for AKI staging. Patients were treated using a multimodal concept that included analgesia and sedation, fluid administration and lung-protective mechanical ventilation, anticoagulation, as well as hemodynamic, antibiotic and diagnostic management as described previously [14]. Continuous dialysis, as required, was technically performed by the hospital’s Department of Nephrology, according to standardized protocols. The observation period was defined from admission on our ICU either to day 30 of hospital stay or death.

### Study groups and end points

The ARDS patients were assigned to two groups (AA genotype vs. AC/CC genotype) depending on the -1364A/C polymorphism in the *AQP5* gene promoter.

The primary end point was an AKI stage ≥ 1 according to the KDIGO Clinical Practice Guidelines for Acute Kidney Injury on day 30 of ICU stay. A secondary end point was the net fluid balance on day 30 of ICU stay.

### DNA-genotyping

DNA was extracted from whole blood using the QIAamp-Kit (QIAGEN, Hilden, Germany). For genotyping the SNP -1364A/C in the *AQP5* promoter, polymerase chain reaction was performed with the forward *AQP5*-SE 5´-GAAACTGCAGGATGAGAGAAAT-3´ and the biotinylated reverse *AQP5*-AS 5´-TCTCTGTTCTCCACCTCTCCA-3´ followed by pyrosequencing, as described previously [13, 14].

### Statistical analysis

The characteristics of the patients at baseline were reported as percentages for categorical variables and as means with standard deviations (± SD) or medians with interquartile ranges (25^th^; 75^th^ percentile) for continuous variables, as appropriate. Categorical variables were compared with chi-square or Fisher’s exact tests, and continuous variables were compared with parametric Student’s t-test or non-parametric Wilcoxon-Mann-Whitney-Test. The potential link between the *AQP5* -1364A/C promoter SNP genotypes and AKI was assessed using a multivariate logistic regression model. The regression model was adjusted for clinically pertinent confounding factors and factors significant in the univariate analysis.

The *AQP5* -1364A/C promoter SNP distributions were tested for deviations from the Hardy-Weinberg equilibrium (exact two-sided P value, significance value 0.05). Explorative comparisons by *AQP5* -1364A/C genotypes (AC/CC vs. AA) were performed for several clinical patient characteristics (Table 1). AC and CC genotypes were combined because of the low frequency (3.7%) of the CC genotype, referring to a dominant model with “A” as a risk allele and “C” as a protective or low-risk allele.

**Table 1:** Baseline characteristics upon ICU admission of patients with pneumonia evoked ARDS stratified for *AQP5* -1364A/C genotypes (n = 136).

Variable	AA n = 93	AC/CC n = 43	P-value
Age *yrs*. (range/± SD)	43.9 (18-77/±15.8)	43.4 (18-66/±13.8)	0.866
Male gender	55 (59%)	24 (56%)	0.715
Body-mass-index (*kg/m*^*2*^)	26.7 (±6.6)	25.9 (± 6.2)	0.558
ARDS severity *			0.489
- mild	17 (18%)	9 (21%)	
- moderate	27 (29%)	16 (37%)	
- severe	49 (53%)	18 (42%)	
**Organ function assessment**			
*Renal*			
- Creatinine concentration *(mg/ml)*	1.53 [0.97–2.94]	1.39 [1.00–1.93]	0.204
- Cont. hemofiltration/dialysis	47 (51%)	18 (42%)	0.346
*-* AKI stage 1	10 (11%)	4 (9%)	0.806
- AKI stage 2	23 (25%)	14 (33%)	
- AKI stage 3	21 (23%)	8 (19%)	
*Circulatory*			
- Mean arterial pressure *(mmHg)*	76 [68–88]	82 [72–88]	0.178
- Cardiac index *(L/min/m*^*2*^*)*	4.1 [2.9–4.9]	3.8 [3.2–4.8]	0.763
- Vasopressor support (n/%)	79 (85%)	34 (79%)	0.395
- Inotropic support (n/%)	36 (39%)	14 (33%)	0.489
- ECMO therapy (n/%)	23 (25%)	14 (33%)	0.340
*Respiratory*			
- Mean airway pressure *(mmHg)*	28 [24–31]	27 [22–31]	0.441
- Lung compliance *(ml/mbar)*	29 [20–41]	28 [18–41.5]	0.854
- Horowitz index *(paO*_*2*_*/F*_*i*_*O*_*2*_*)*	89.5 [68–137]	113 [85–179]	0.129
- Lung injury score	3.3 (±0.5)	3.3 (±0.5)	0.867
*Inflammatory*			
- C-reactive protein concentration (*mg/dl)*	20.3 [14.8–27.4]	18.5 [8,5–23.8]	0.091
- Procalcitonin concentration (*ng/m*l)	10.4 [1.0–55.5]	5.0 [1.0–29.0]	0.324
- Leukocyte concentration (*10^*9*^*/l*)	14.0 [9.0–23.1]	16.1 [11.4–23.3]	0.283
*Hepatic*			
- Total bilirubin concentration (*mg/dl)*	1.0 [0.6–2.0]	0.9 [0.5–1.8]	0.633
- AST *(U/l)*	62 [33–157]	49 [31–114]	0.326
- ALT *(U/l)*	36 [19–74]	31 [19–75]	0.872
*Organ dysfunction scores*			
- SAPS II	51.7 (±17.2)	49.9 (±15.9)	0.566
- SOFA	13.4 (±4.9)	13.8 (±6.1)	0.716
**Outcome**			
- ICU stay *(days)*	27.0 [±25.8]	25.0 [±14.6]	0.631
- Duration of mechanical ventilation *(days)*	14.6 [±9.1]	16.4 [±8.3]	0.271
- Ventilator free days	9.5 (±9.6)	10.7 (±9.0)	0.504
- RRT free days	7.7 (±9.4)	10.6 (±9.3)	0.099
- 30-days mortality	35 (37.6%)	6 (13.9%)	0.005

Data are presented as n (%); means (± SD), and medians (25th, 75th percentile)

*ARDS severity was stratified according to Berlin definition [16]

ECMO: extracorporeal membrane oxygenation; Horowitz index: paO_2_/F_i_O_2_; AST: Aspartate aminotransferase; ALT: Alanine aminotransferase; SAPS II: Simplified Acute Physiology Score; SOFA: Sepsis-related Organ Failure Assessment score, RRT: Renal replacement therapy

All analyses were conducted at a two-sided alpha error level of 5%. All analyses were performed using SPSS (version 24, IBM, Chicago, IL, USA). For graphical presentations GraphPad Prism 7 (Graph-Pad, San Diego, CA, USA) was used.

## Results

The observed 30-day survival of the entire cohort was 69% and the median duration of the ICU stay was 21 days [11; 34 days]. The baseline characteristics of the 136 patients stratified for their *AQP*5 promoter SNP genotypes and for survivors vs. non-survivors are presented in Tables 1 and 2, respectively.

**Table 2:** Baseline characteristics upon ICU admission of patients with pneumonia evoked ARDS as stratified for 30-day survival and non-survival (n = 136).

Variable	Survivors n = 95	Non-Survivors n = 41	P-value
Age *yrs*. (range/± SD)	43.2 (18-77/±15.2)	44.9 (18-66/±15.1)	0.866
Male gender	54 (57%)	25 (61%)	0.654
Body-mass-index *(kg/m*^*2*^*)*	26.9 (±6.4)	25.5 (± 6.5)	0.259
ARDS severity *			0.655
- mild	20 (21%)	6 (14%)	
- moderate	30 (32%)	13 (32%)	
- severe	45 (47%)	22 (54%)	
**Organ function assessment**			
*Renal*			
- Creatinine concentration *(mg/ml)*	1.31 [0.88–2.61]	1.69 [1.34–2.79]	0.038
- Cont. hemofiltration/dialysis	43 (45%)	22 (54%)	0.368
*-* AKI stage 1	8 (8%)	6 (15%)	0.143
- AKI stage 2	32 (34%)	6 (15%)	
- AKI stage 3	17 (12%)	11(27%)	
*Circulatory*			
- Mean arterial pressure *(mmHg)*	82 [72–89]	72.5 [68–81]	0.034
- Cardiac index *(L/min/m*^*2*^*)*	4.1 [3.2–4.8]	3.8 [2.7–5.2]	0.647
- Vasopressor support (n/%)	80 (84%)	33 (80%)	0.595
- Inotropic support (n/%)	31 (33%)	19 (46%)	0.128
- ECMO therapy (n/%)	25 (26%)	12 (29%)	0.723
*Respiratory*			
- Mean airway pressure *(mmHg)*	28 [23–31]	28 [24–31]	0.899
- Lung compliance *(ml/mbar)*	28 [20–42]	29 [17–40]	0.623
- Horowitz index *(paO*_*2*_*/F*_*i*_*O*_*2*_*)*	105 [69.5–176]	90 [72–117]	0.297
- Lung injury score	3.3 (±0.5)	3.2 (±0.5)	0.585
*Inflammatory*			
- C-reactive protein concentration (*mg/dl)*	18.0 [10.3–27.3]	19.6 [7.6–24.7]	0.811
- Procalcitonin concentration (*ng/m*l)	5.8 [0.6–35.4]	10.5 [3,8–47.3]	0.110
- Leukocyte concentration (*10^*9*^*/l*)	15.9 [10.1–23.6]	12.7 [8.8–17.2]	0.102
*Hepatic*			
- Total bilirubin concentration (*mg/dl)*	0.9 [0.5–1.5]	1.3 [0.7–3.0]	0.011
- AST *(U/l)*	52 [31–110]	111 [44–360]	0.007
- ALT *(U/l)*	31 [18–58]	39 [20–132]	0.043
*Organ dysfunction scores*			
- SAPS II	48.4.7 (±16.4)	57.3 (±16.0)	0.004
- SOFA	12.8 (±5.7)	15.4 (±3.4)	0.011
**Outcome**			
- ICU stay *(days)*	29.9 (±24.8)	18.2 (±14.8)	0.006
- Duration of mechanical ventilation *(days)*	16.9 (±8.6)	11.1 (±8.2)	<0.001
- Ventilator free days	11.6 (±8.9)	5.9 (±9.2)	0.001
- RRT free days	10.1 (±9.5)	5.2 (±8.2)	0.005

Data are presented as n (%); means (± SD), and medians (25th, 75th percentile)

*ARDS severity according to Berlin definition [16]

ECMO: extracorporeal membrane oxygenation; Horowitz index: paO_2_/F_i_O_2_; AST: Aspartate aminotransferase; ALT: Alanine aminotransferase; SAPS II: Simplified Acute Physiology Score; SOFA: Sepsis-related Organ Failure Assessment score; RRT: Renal replacement therapy

Regarding distribution of the genetic variations according the Hardy-Weinberg equilibrium of the *AQP5* SNPs we observed 93 AA genotypes (expected: 92.2), 38 for the AC genotype (expected: 39.5), and 5 for the CC genotype (expected: 4.2). Accordingly, there was no deviation from the Hardy-Weinberg equilibrium (p = 0.655).

**Fig 1:**
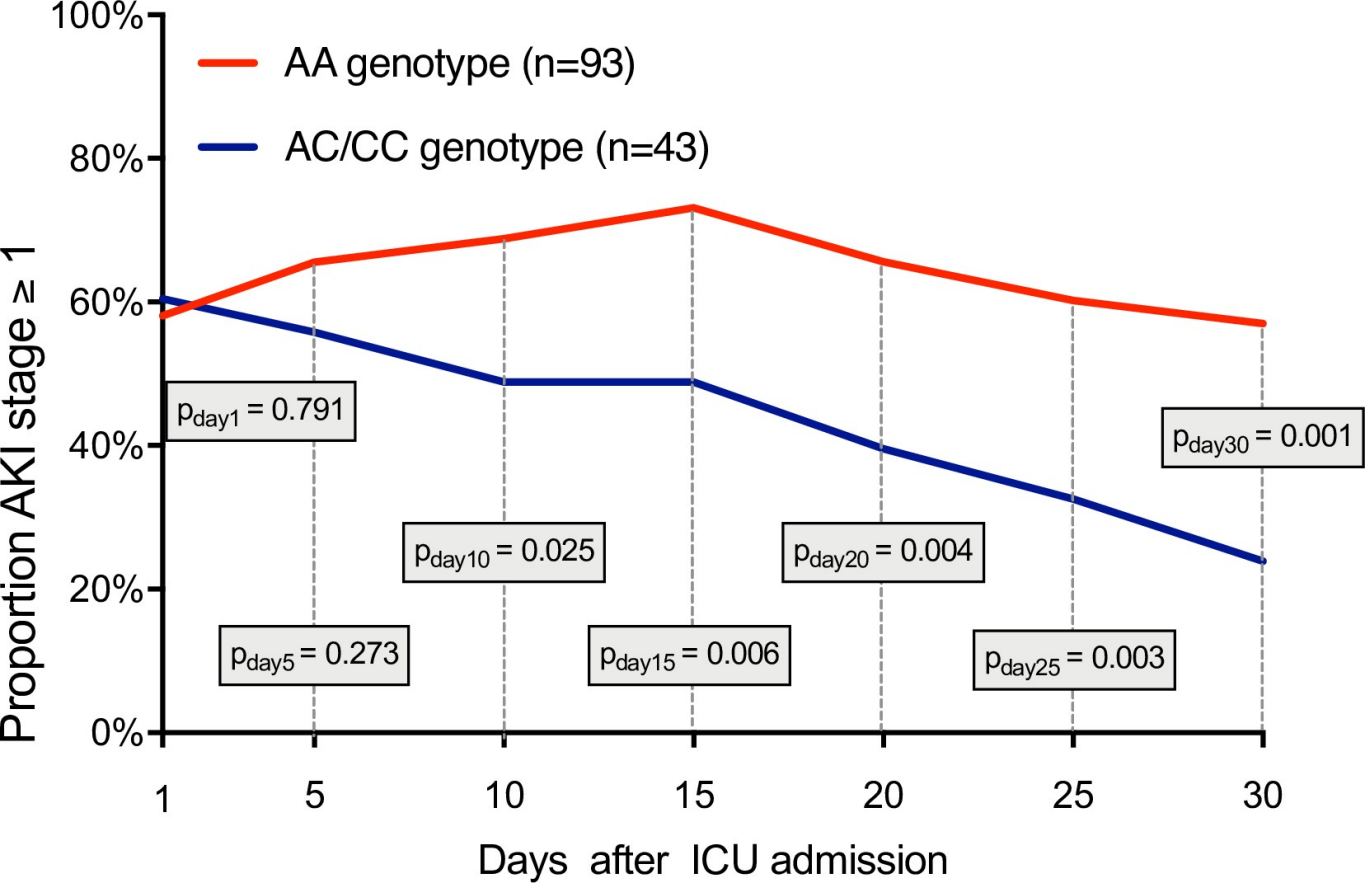
Proportion of all patients (n = 136) with acute kidney injury (AKI stage ≥ 1 according to KDIGO criteria) in AA und AC/CC genotypes of the *AQP5* -1364A/C promoter SNP. Measurements on day 1, 5, 10, 15, 20, 25 and 30.

Upon ICU admission, AA genotypes (58%) and AC/CC genotypes (60%) showed comparable frequencies of acute kidney injury with an AKI stage ≥ 1 (p = 0.791; Fig 1).

No differences were found between AA and AC/CC genotypes regarding serum creatinine (p = 0.204), necessity for continuous hemofiltration/-dialysis (p = 0.346), vasopressor support (p = 0.395), simplified Acute Physiology Score II (p = 0.566) or Sequential Organ Failure Assessment score (p = 0.716; Table 1). Moreover, there was no genotype-dependent pattern for infection type (p = 0.495) or primary diagnosis at hospital admission (p = 0.867). However, despite similarity in these variables the AA genotype was associated with an increased mortality (p = 0.005).

A significantly higher rate of AKI persisted in AA genotypes from day 10 and on (p = 0.025; Fig 1). On day 30, 57% of AA genotype patients still had an AKI stage ≥1, compared to only 24% in AC/CC genotypes (p = 0.001; Fig 1). Furthermore, the AA genotype of the *AQP5* promoter SNP was an independent risk factor for an AKI with an estimated odds ratio of 3.35 (CI-95%: 1.2–9.0; p = 0.017; Table 3).

**Table 3:** Factors independently associated with AKI after adjustment for confounders (stepwise logistic regression).

(Co) variable	Multivariate					
	Initial			Restricted		
	p-value	OR	95%- CI	p-value	OR	95%- CI
Aquaporin 5 –1364A/C genotype						
- AC/CC	-	1		-	1	
- AA	0.025	3.203	1.516–8.874	0.017	3.350	1.244–9.021
Age [per year]	0.045	1.033	1.000–1.067	0.048	1.020	1.000–1.046
Dialysis [no]	-	1				
Dialysis [yes]	0.302	0.584	0.210–1.622			
ECMO [no]	-	1				
ECMO [yes]	0.122	0.404	0.132–1.235			
Cumulative fluid balance on day 30						
-2.5L - +2.5L	-	1	1			
< -2.5L	0.432	0.639	0.209–1.953			
> +2.5L	0.398	1.728	0.486–6.139			

OR: Odds ratio point estimates, 95% CI, and p-values (two-sided) are reported. Homer-Lemeshow statistics were as follows: κ^2^ = 13.2; p = 0.104

**Fig 2:**
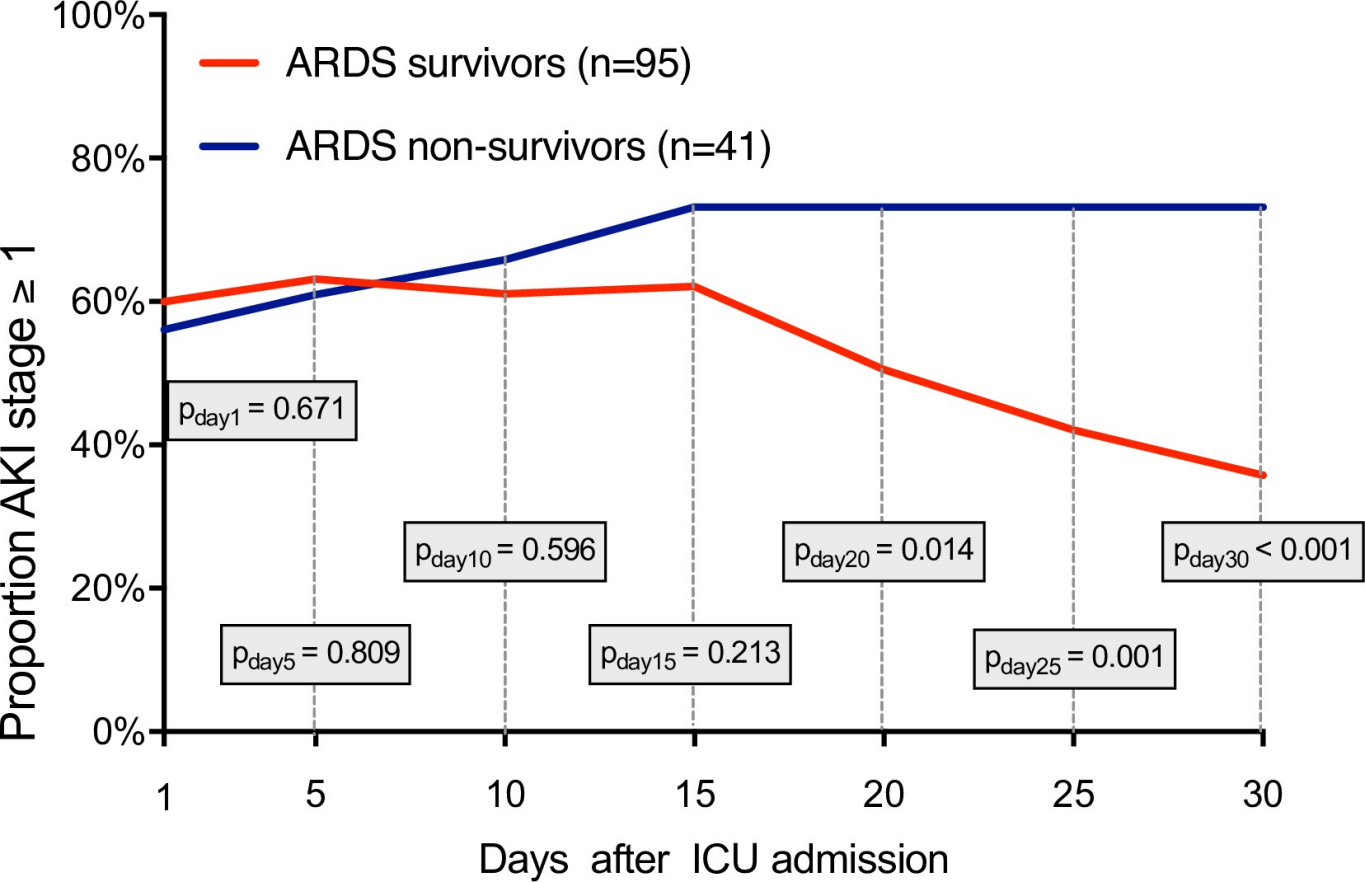
Proportion of all patients (n = 136) with acute kidney injury (AKI stage ≥ 1 according KDIGO criteria) stratified for survivors and non-survivors. Measurements on day 1, 5, 10, 15, 20, 25 and 30.

Comparing the prevalence of AKI on admission between 30-day survivors (60%) and non-survivors (56%) we also found no significant difference (p = 0.671; Fig 2).

However, with regard to organ functions (Table 2), non-survivors showed greater serum creatinine concentrations (p = 0.038), greater total bilirubin concentrations (p = 0.011), a greater aspartate aminotransferase (p = 0.007) and alanine aminotransferase activity (p = 0.043), as well as lower mean arterial pressures (p = 0.034) and fewer ventilator free days (p = 0.001). On day 30 seventy-three per cent of non-survivors but only thirty-six per cent of survivors had an AKI stage ≥1 (p<0.001; Fig 2).

**Fig 3:**
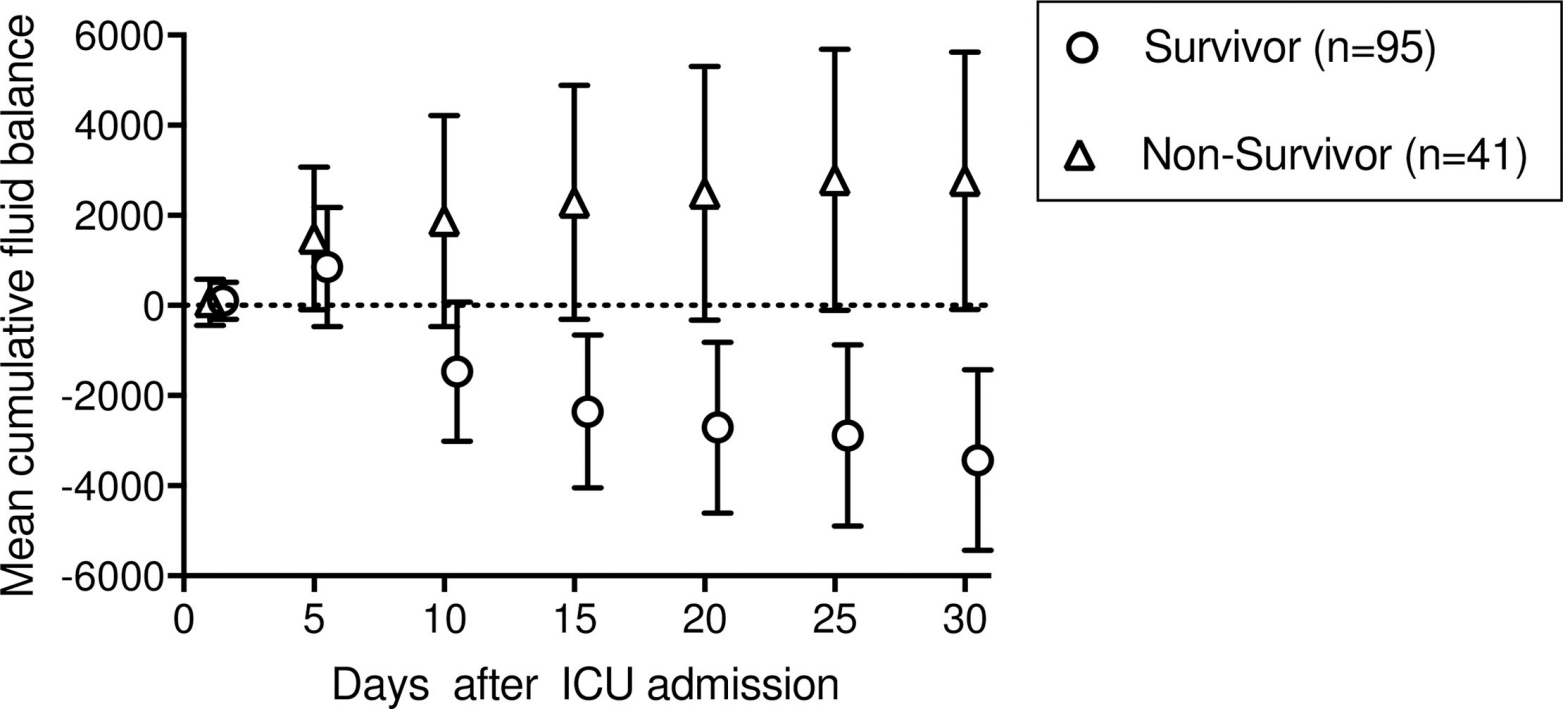
Mean cumulative fluid balance with 95% CI for survivors and non-survivors until ICU day 30. The fluid balance in patients who died was markedly greater than in the survivors.

**Fig 4:**
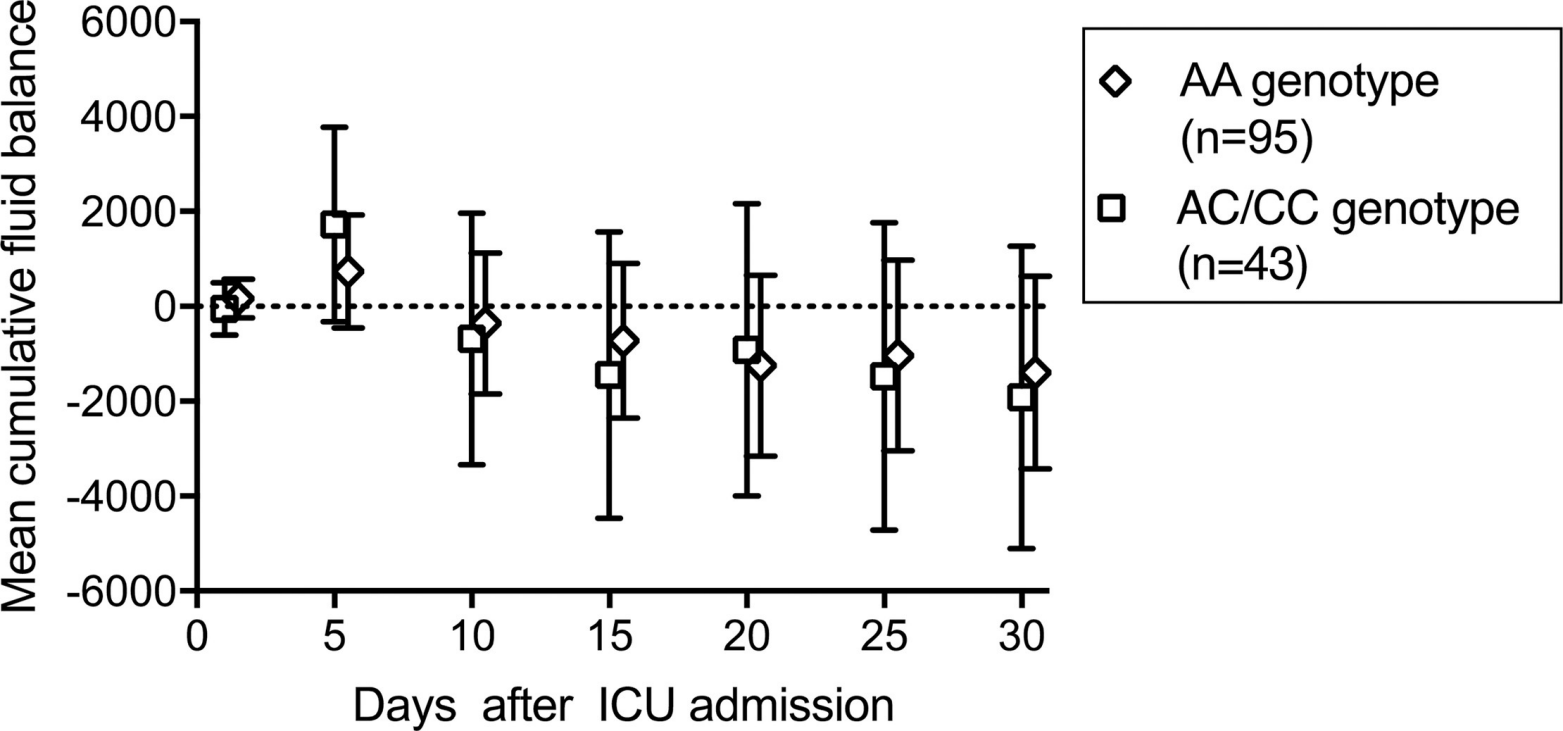
Mean cumulative fluid balances with 95% CI stratified for AA and AC/CC genotypes of the *AQP5* -1364A/C promoter SNP until ICU day 30. The fluid balance showed no difference between the *AQP5* genotypes.

The cumulative net fluid balance on days 1, 5, 10, 15, 20, 25, and 30 stratified for survivors and non-survivors and for AA genotypes and AC/CC genotypes are depicted in Figs 3 and 4, respectively.

A significant difference of the net fluid balance of survivors and non-survivors was observed from day 10 and on (p = 0.017). The cumulative net fluid balance on day 30 for survivors was -3.4 L (95%CI: -1.4 to -5.4) but +2.8L (95%CI: +0.1 to +5.6 for non-survivors (p = 0.001; Fig 3) but was not different between AA genotypes (-1.4L; 95%CI: +0.6 to -3.4) and AC/CC genotypes (-1.9L; 95%CI: +1.2 to -5.1) (p = 0.959; Fig 4). Furthermore, we found a negative cumulative fluid balance on day 30 in almost all patients without AKI (95%-CI: -4.6L - -0.4L), whereas patients with an AKI stage ≥1 showed a trend to more positive cumulative fluid balance on day 30 (95%-CI: -3.1L - 1.6L), but without statistical significant difference (p = 0.078; S1 Fig).

## Discussion

We found a significant association between the *AQP5* -1364A/C promoter SNP genotype and recovery from AKI during treatment of patients suffering from pneumonia evoked ARDS. Furthermore, the AA genotype is a strong and independent risk factor for AKI with an estimated odds-ratio of 3.35. While resolution of AKI was strongly linked to the *AQP5* genotype it was not related to the patients`net fluid balance suggesting that these genotype dependent effects are mediated by pathways other than those related to renal excretory function.

Despite advances in therapy, the morbidity and mortality of ARDS and AKI still remains unacceptably high, especially if multiple organ failures develop concurrently [4, 5] and persist longer [6, 18]. A growing evidence points to deleterious interactions between lung and kidney, explaining at least in part the high incidence and mortality in patients suffering from ARDS and AKI [4, 19]. Experimental studies have revealed increased airway and alveolar pressure, fluid overload and systemic inflammation as potential pathways evoking kidney dysfunction [20–24]. Furthermore, excessive positive-pressure ventilation affects systemic inflammation and immune cell migration [25] with higher frequencies of an inflammation-dependent AKI [4, 19, 26].

However, notwithstanding a large number of reports suggesting an interaction between respiratory failure, inflammation and AKI, only few studies have addressed genetic risk factors in ARDS evoked renal dysfunction. In the present study, we reveal by demonstrating a significant higher rate of AKI in AA genotypes on day 30 that the functionally relevant *AQP5* promoter polymorphism impacts on recovery of kidney function. In fact, the AA genotype turned out to be a strong, significant and independent risk factor for AKI during the course of pneumonia evoked ARDS.

While the exact mechanisms cannot be pinpointed by our study, a few speculations can be made. Many investigators reported an association of fluid balance with ARDS mortality [27, 28]. This is in full conformity with our data since we found that a negative fluid balance from day 10 and on is associated with a significantly lower 30-days mortality. However, since the *AQP5* promoter polymorphism did not impact on cumulative net fluid balance, the genotype dependent resolution of AKI and survival are probably due to mechanisms unrelated to *APQ5* effects on renal excretory function and fluid balance. In this context, recent experimental studies of *AQP5* expression *in-situ* in kidneys of mice, rats and humans failed to reveal an impact of *AQP5* in active transepithelial fluid absorption under normal conditions [29] or in acute lung injury [8].

Decreased recovery from acute kidney injury and increased mortality in AA genotypes in ARDS is more likely mediated by increased pulmonary inflammation than due to the role of aquaporins in water transport or fluid balance. Previous studies have suggested that AQP5 seems to be a key protein of inflammation in severe sepsis and ARDS [12, 30]. In this context, wild type mice show an increased neutrophil migration into the lung and greater mortality compared to AQP5-knock-out mice after intraperitoneal lipopolysaccharide injection [12]. Additionally, target-oriented migration of human neutrophils across a membrane is significantly faster and more profound with increased AQP5 expression in the AA genotype of the AQP5 -1364A/C SNP [12]. Given the presence of a large population of activated leukocytes within the inflamed lung potentially also impacts on the kidneys by a harmful lung-kidney organ crosstalk [31] with a recent study identifying markedly similar expression of 109 important proinflammatory genes occurring homogenously in both lung and kidneys during an AKI [32].

### Limitations

Our study has limitations. First, the observational design and lack of histologic and mechanistic examinations precludes verification about causality and mechanisms. Furthermore, more detailed data regarding causes of death, side effects, and longer follow up period may have expanded insights but were unavailable. Therefore, associations between the AQP5 promoter SNP and AKI are limited to day 30 predictions. Second, we did not consider the long-term renal recovery of survivors beyond the 30-days observation period. Third, unrecognized selection bias, inherent to many genetic association studies, cannot be excluded entirely. Since our study was conducted in patients of European-Caucasian descent, findings cannot be generalized to subjects of other ancestries. However, the single center nature of this study may be an advantage as it limits variation in therapeutic protocols among institutions.

### Conclusion

In pneumonia evoked ARDS, the AA genotype of the *AQP5* -1364A/C promoter SNP is associated with a decreased recovery rate from acute kidney injury and this is independent from the patients net fluid balance. This suggests that mechanisms other than *AQP5* effects on renal water excretion and fluid balance are responsible.

## Supporting information

S1 Fig Mean cumulative fluid balance with 95% CI stratified for patients with AKI (AKI stage ≥ 1) and without AKI until ICU day 30. Measurements on day 1, 5, 10, 15, 20, 25, and 30.
(PDF)

S2 Fig Proportion of all patients stratified for AKI stage 1, 2, and 3 according to KDIGO criteria. Measurements on day 1, 5, 10, 15, 20, 25, and 30.
(PDF)

S1 File Raw data set. (XLSX)
