# Supplementary material for: The aquaporin 5 -1364A/C promoter polymorphism impacts on resolution of acute kidney injury in pneumonia evoked ARDS
Source: PLoS One. 2018 Dec 5;13(12):e0208582. doi: 10.1371/journal.pone.0208582 (PMC6281272; doi:10.1371/journal.pone.0208582)

S2 Figure. Proportion of all patients stratified for AKI stage 1, 2, and 3 according to KDIGO criteria. Measurements on day 1, 5, 10, 15, 20, 25, and 30.

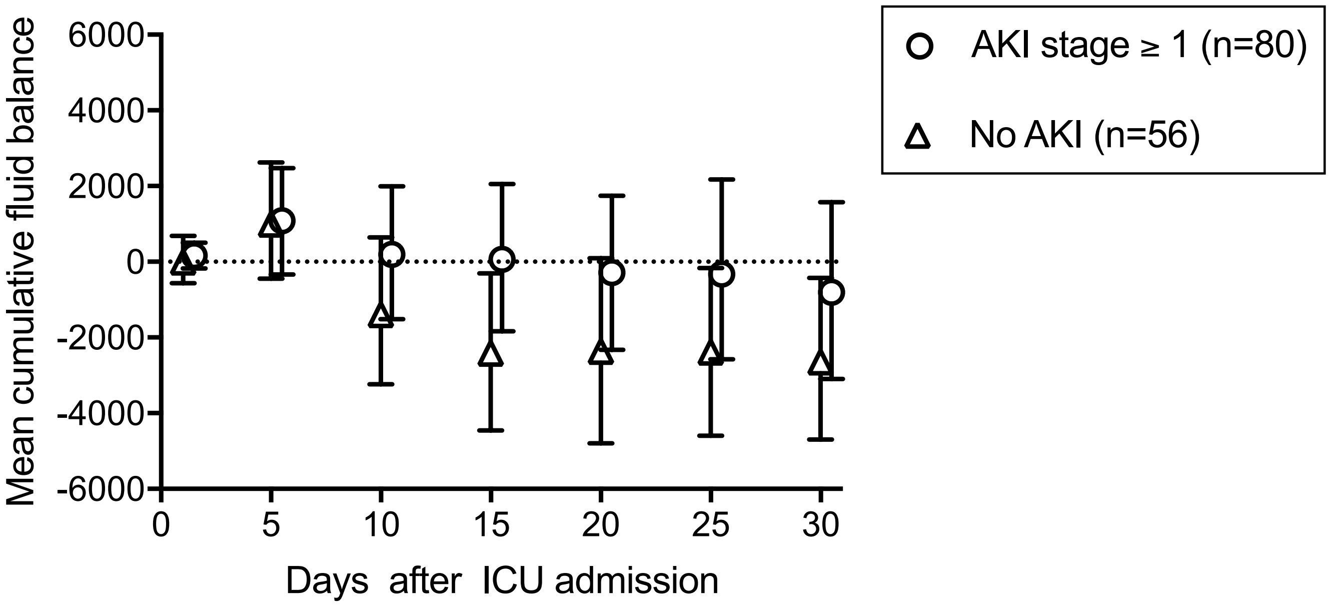

Supplement: S1 Fig — Measurements on day 1, 5, 10, 15, 20, 25, and 30. (PDF) [file pone.0208582.s001.pdf]
